# Reconstructing horizontal gene flow network to understand prokaryotic evolution

**DOI:** 10.1098/rsob.220169

**Published:** 2022-11-30

**Authors:** Soham Sengupta, Rajeev K. Azad

**Affiliations:** ^1^ Department of Biological Sciences and BioDiscovery Institute, University of North Texas, Denton, TX 76203, USA; ^2^ Department of Mathematics, University of North Texas, Denton, TX 76203, USA

**Keywords:** horizontal gene transfer, prokaryotes, gene clustering, phyletic pattern, network

## Abstract

Horizontal gene transfer (HGT) is a major source of phenotypic innovation and a mechanism of niche adaptation in prokaryotes. Quantification of HGT is critical to decipher its myriad roles in microbial evolution and adaptation. Advances in genome sequencing and bioinformatics have augmented our ability to understand the microbial world, particularly the direct or indirect influence of HGT on diverse life forms. Methods for detecting HGT can be classified into phylogenetic-based and parametric or composition-based approaches. Here, we exploited the complementary strengths of both the approaches to construct a high confidence horizontal gene flow network. Our network is unique in its ability to detect the transfer of native genes of a genome to genomes from other taxa, thus establishing donor and recipient organisms (taxa), rather than through a post hoc analysis as is the practice with several other approaches. The scale-free horizontal gene flow network presented here provides new insights into modes of transfer for the exchange of genetic information and also illuminates differential gene flow across phyla.

## Graphical abstract

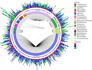

## 1. Introduction

Horizontal gene transfer (HGT), an evolutionary mechanism enabling transfer of genetic material through means other than vertical inheritance (parents to progenies), has an immense influence on prokaryotic evolution [1–3]. Though long recognized as an important factor in microbial evolution, its extent and impact on organismal evolution were not established until recently, when many studies leveraged the large-scale genomic data available in public repositories to understand microbial evolution using different approaches [4–8]. Rapid growth of genomic databases, with now tens of thousands of completely sequenced prokaryotic genomes or genome assemblies made available for analysis, has enabled reassessment of HGT at an unprecedented scale and at a much higher resolution in different prokaryotic lineages. Recent studies have reaffirmed the profound contribution of HGT in shaping prokaryotic genomes, with several suggesting that HGT has occurred at least once during evolution of almost all genes, with probably only a handful of genes still immune to it [3,9].

Methods for cataloguing HGT events have primarily relied on phylogenetic approaches. Although HGT obfuscates the tree metaphor of evolution of life, phylogenetic trees have been most used in HGT inference based on the premise that organismal evolution can still be reconciled at best in the form of a tree and HGT, therefore, may be detected as an aberration against this reconciled norm of evolution. Inference of organismal phylogeny is non-trivial and therefore a number of approaches have been taken, including those that are based on compositional signatures [10], conserved marker sequence alignments [11] or alignments of artificially concatenated conserved orthologues [12,13]. Although incongruence between single-gene trees and the ‘consensus’ species tree is often invoked to infer horizontal inheritance, specifically ancient transfer events, this approach is constrained by factors such as gene loss, biased mutation rates, improper clade selection, long-branch-length attraction and segregation of paralogues. An alternative tree-independent phylogenetic approach is based on search for unusual phyletic patterns in pairwise comparisons of closely related genomes [14–16]. Genes with limited phylogenetic distribution—those absent from the genomes of close relatives—are inferred to be horizontally acquired genes. This, however, requires multiple strains of closely related species. Lineage-specific gene loss and arbitrarily chosen phylogenetic distance may further confound the interpretation of results from such analysis. Furthermore, the ‘orphan’ alien genes (those lacking homologues) are not possible to detect using these approaches. Above all, the success of phylogenetic methods solely depends on the breadth and depth of the sequence database.

An alternative approach, often called parametric or composition-based methods, exploits the atypical compositional features of horizontally acquired genes, such as unusual GC content, oligonucleotide composition or codon usage pattern [8,17–23]. These atypical features reflect the compositional biases of donors and can thus be identified as distinct signals against a recipient genome background. This approach allows inference of foreign genes without multiple genome comparisons and thus is free from numerous vagaries of the phylogenetic approaches [24]. However, as time passes, as a consequence of directional mutational pressure, the acquired genes ameliorate their composition to that of the recipient genome [25]. Parametric methods may, therefore, fail to detect ancient transfers. Lawrence and Ochman have shown that most alien genes are recent acquisitions [20,25,26]. Most acquired genes do not provide long-term benefits and are eventually lost. Therefore, at a certain snapshot of time, most resident alien genes could likely be recent acquisitions. Parametric methods have frequently been invoked to estimate the genome-scale impact of recent HGTs.

Azad & Lawrence [23] have shown that by grouping genes that are compositionally similar to each other through a gene clustering approach, foreign genes could be identified more robustly, in contrast to assessment of atypicality of each gene against the genome background that is often non-trivial to establish. Their method, Jenson-Shannon Codon Bias (JS-CB), was demonstrated to be efficient in localizing even antibiotic resistance and pathogenicity islands in methicillin-resistant *Staphylococcus aureus* (MRSA) genomes [27]. Although these and later developments enabled reliable estimation of acquired genes in prokaryotes, relatively much less is known about the pattern of differential gene flow among prokaryotes. Identification of genes of unusually high similarity in otherwise distant taxa has been investigated using alignment-based methods such as BLAST; however, without regard to the donors and recipients or the direction of gene flow [28,29]. Phyletic pattern-based approach infers foreign genes based on pattern of the presence or the absence of genes in close relatives and is not focused on determining the potential donors of acquired genes. Phylogenetic tree-based methods infer donors and recipients and thus the direction of gene flow, however, their inferences could be confounded by the numerous vagaries of these methods. Note that these methods assume the tree form of evolution, which may not be true specifically in prokaryotes due to frequent HGT. To address this conundrum, methods that enable visualization of the evolution as a network rather than a tree have been proposed [28–32].

For understanding the pattern of horizontal gene flow, phylogenetic methods have often been invoked and therefore, a large body of literature exists on the usage of phylogenetic approaches in this context. Composition-based methods have sparingly been used in deciphering the pattern, although successive studies have highlighted the complementary strengths of these methods [8,24,33]. Although these methods may not be able to detect ancient gene transfers due to the amelioration of composition of the acquired genes to the native genome composition with the passage of time, they could be used for assessing genome-scale impact of recent HGTs. Note that ancient transfers with feeble or muddled evolutionary signals could be challenging to detect for any methods, including phylogenetic approaches. As mentioned, most alien genes in a prokaryotic genome are likely recent acquisitions, and therefore, the parametric or composition-based methods are powerful tools for quantifying the horizontally acquired DNAs. Another prominent advantage of using these methods is their ability to detect ‘orphan’ alien genes; these genes with no homologues cannot be analysed using comparative or phylogenetic approaches. Recently acquired orphan genes could, however, be identified based on their anomalous compositional characteristics using parametric methods. These orphan alien genes might have originated from organisms whose genomes are yet not sequenced or could have rapidly evolved or undergone substantial rearrangements rendering them unalignable and therefore, intractable with phylogenetic approaches. Parametric methods, on the other hand, can still infer the presence of orphan alien genes in prokaryotic genomes based on composition that can account for subtle evolutionary signals encoded in short oligomers.

With these considerations and to leverage the complementary strengths of parametric methods, we revisited the JS-CB method, with the objective to gain new insights into the pattern of horizontal gene flow among prokaryotes. We selected JS-CB for the following reason. In contrast to other parametric methods, most of which classifies genes into only two classes, namely, native and alien, JS-CB segregates alien genes into multiple classes in addition to identifying a single native class, with each class harbouring genes of similar composition. Each alien class could thus be representing a potential donor source. We exploited this attribute of JS-CB to construct a gene exchange network based on compositional (codon usage) similarity of gene classes across genomes.

Pairwise comparison of gene classes across genomes, within the same hypothesis testing framework established in JS-CB for gene clustering, led to the generation of a gene-clustering-based network. Because compositional atypicality, including atypical codon usage bias, can arise for reasons other than horizontal gene acquisition, each atypical cluster was assessed for enrichment of marker genes typically associated with horizontal DNA mobilization (such as those encoding transposase, integrase and recombinase, and prophage as well as plasmid) and for unusual phyletic pattern of genes harboured by it. The atypical clusters supported by either of these lines of evidence were inferred alien and subjected to further downstream analysis to unravel the pattern of gene flow among prokaryotes. The proposed network is unique and novel in manifold ways and complements phylogenetic approaches. Because the backbone (native) genome of an organism is identified as a distinct large cluster, inter-genome cluster comparison allows to identify alien clusters from different organisms (taxa) that are similar to the native cluster of an organism (taxon) of interest. This aids in inferring the direction of flow from the latter (donor) to the former (recipients). Note that phylogenetic tree-based approaches also reveal the polarity (donor and recipient) but without regard to whether a gene has been acquired from native or alien part of a donor genome. The proposed approach allows to catalogue genes that have arrived from the backbones (native regions) of donor genomes. In addition, in conjunction with the tree-based approaches, it can be used to infer the flow of genes from alien regions of donor genomes to the recipient genomes. Our approach thus allows visualization of mobility of both native and alien parts of donor genomes and thus uncovers hitherto unknown aspects of gene flow among prokaryotes. Furthermore, as a donor is likely to contribute multiple genes to a recipient, this is appropriately accounted for through gene clustering before constructing the network of gene flow. This allows visualization of mobility of groups of genes of potentially shared ancestry in the form of a gene clustering network, in contrast to gene-level flow visualization rendered by phylogenetic tree-based approaches. In what follows, we describe the methodology for construction of gene flow network, results from our analysis of the network including the properties of this network, patterns of gene exchange among prokaryotic lineages, and functions of the acquired genes, and finally, we discuss the novel aspects of our study and future directions.

## 2. Methods

### 2.1. Prokaryotic genomes

The complete genome sequences of over 700 representative bacteria and archaea were downloaded from NCBI FTP database. The accession number and taxonomic details are provided in electronic supplementary material, table S1. Gene sequences were retrieved from the respective genomes using gene coordinates provided in the annotation files.

### 2.2. Gene clustering using JS-CB

For identifying genes originating from distinct donor sources, we employed the Jenson-Shannon Codon Bias (JS-CB) approach as described in [23]. JS-CB is a gene clustering method that identifies putative horizontally acquired genes by first grouping genes of similar codon usage biases into distinct clusters [23]. JS-CB uses the Jensen–Shannon (JS) divergence measure [34,35] to assess the difference in codon usage bias between two genes. Genes with similar codon usage bias are grouped together using an agglomerative hierarchical clustering procedure. JS-CB begins with all individual genes as single-gene clusters, followed by pairwise comparison of the clusters. The two most similar gene clusters (in terms of JS divergence) are merged iteratively within a statistical hypothesis framework. If the *p*-value for a gene cluster pair, computed based on an analytic approximation of the probability distribution of JS divergence, is less than a preset significance level, the gene clusters are deemed different, otherwise they are merged. The process is performed recursively resulting in clusters of genes with similar codon usage biases. Genome positional information of genes was also considered explicitly to merge clusters following creation of pure clusters at stringent thresholds [23]. Variants of this prioritize clustering of proximal similar genes or genomic segments (e.g. [4]); here contiguous similar genes (segments) are grouped first at a stringent setting, followed by grouping of clusters recursively at a relaxed setting within the statistical hypothesis testing framework. This was used here, with significance threshold set at 10^−4^ for clustering of contiguous similar genes and at 10^−8^ for subsequent grouping of similar clusters recursively. The largest cluster represents the native genes, while the numerous smaller clusters may harbour putative alien genes. The performance of this approach in identifying alien genes or genomic segments has previously been reported in [4,23,24,27].

Because compositional atypicality (unusual codon usage bias) of genes resident in smaller clusters may also arise due to factors other than horizontal acquisition (e.g. highly expressing ribosomal protein genes display atypical codon usage and may be assigned to a distinct atypical cluster), we performed marker gene enrichment and phyletic pattern analyses for atypical clusters in order to compile a high confidence set of putatively alien clusters for each genome, as described below.

### 2.3. Marker gene enrichment analysis

Marker gene enrichment analysis was performed on each cluster using HMMER [36] against a custom PFAM database [37] constructed as described in [8]. Briefly, the custom database was built by performing search for genomic island (GI) specific markers such as, transposase, integrase, prophage, recombinase and plasmid, followed by building a profile HMM for each marker gene family. Additionally, the database was manually curated to eliminate any profile HMMs not representing GI markers. This resulted in a local database of approximately 450 profile HMMs representing marker gene families [8] (https://github.com/sohamsg90/Gene-flow-network). Genes belonging to an atypical cluster were compared against this database and those with ‘hits’ in the database with expect value of 0.01 or less [38] were annotated as marker genes. For each atypical cluster harbouring marker genes, fold enrichment against the respective genome background, as well as against the respective largest (native) cluster, was computed. Clusters with significant enrichment (with respect to native), inferred using hypergeometric test at significance level of 0.05, were annotated as alien clusters. Other atypical clusters that lack marker gene enrichment needed further evaluation as it is plausible that horizontally acquired marker genes may have been lost in the course of evolution or some acquired clusters may just lack the markers. We subjected these clusters to phyletic pattern analysis as described below.

### 2.4. Phyletic pattern analysis

APP (Alienness by Phyletic Pattern; https://github.com/sohamsg90/APP-Alienness-by-Phyletic-Pattern) [39] was employed to examine phylogenetic distribution of each gene in the atypical clusters that lacked enrichment of marker genes. APP employs BLAST [40] to perform phyletic pattern analysis that entails examining the presence or absence of a gene of interest from a genome in the closely related genomes, from lower to higher taxonomic levels (species to family). For each compositionally atypical cluster lacking marker gene enrichment, if a majority of the resident genes displayed atypical phyletic pattern, the cluster was annotated alien. Otherwise, it is labelled as native.

Phyletically aberrant gene inference for a gene of interest is based on the distribution of the gene within the species, genus and family groups it belongs to. If the query gene was found absent or sporadically present in the genomes of close relatives within the species group, it was inferred phyletically aberrant. If not found phyletically aberrant at the species level, gene distribution was examined at higher taxonomic levels (up to family level). Specifically, if a query gene was found to be present in only 30% of the genomes of the species group it belongs to, it was deemed phyletically aberrant. However, if the gene was found to be well distributed or present in more than 80% of the genomes, we assessed its phyletic pattern in the genomes belonging to its genus group. Note that sequence alignment using BLAST was used to infer sequence homology and thus the presence or the absence of genes (query coverage of 70% was used across all taxonomic ranks, and, per cent identity cutoff was successively relaxed from species to family: 60% at species, 50% at genus and 25% at family levels). If the gene was not found phyletically aberrant at the genus level, that is, was found present in more 80% of the genomes belonging to the genus group, its distribution was observed in the genomes of the corresponding family group. Note that phyletic patterns were assessed at higher taxonomic levels to infer ancient transfers, for example, those that happened in the common ancestor of a species group are expected to be well distributed in the genomes of the species group but will not be so in other species from the same genus (readers are referred to the documentation at APP's GitHub website for further details and instructions for its implementation).

### 2.5. Gene flow network construction

Once the native and alien clusters were established for all genomes, pairwise cluster comparisons were performed across genomes to build a gene cluster-based network. Following assignment of an identity number (ID) to each cluster (e.g. G5_Cl_1 denotes cluster 1 belonging to genome 5, electronic supplementary material, tables S2–S3), pairwise comparisons of the clusters across genomes were performed within the same hypothesis testing framework as established in the JS-CB gene clustering algorithm. Pairs of similar clusters in different genomes were thus identified and plotted using Cytoscape [41], with clusters as nodes and connections between clusters signifying their similarity as edges.

Two types of connections were apparent in this network: native–alien connection indicating similarity between native cluster of a genome and an alien cluster from a different genome, and alien–alien connection indicating similarity of two alien clusters originating from different genomes. The former connection in the network has arisen because of transfer of native genes from one genome (taxon) to another, whereas the latter connection indicates transfer of alien genes from a genome (taxon) to another.

### 2.6. Evaluation of goodness of fit for power-law distribution

In a scale-free network, the degree distribution, defined as *P_k_*, follows a power law *P_k_* ∼ *k*^−*α*^, where *k* is the degree and *α* is the degree exponent value that typically satisfies 2 < *α* < 3. The degree of a node is the number of connections it has with other nodes in a network. To evaluate the goodness of fit of degree distribution for our network to a power-law distribution, we compared it with other distributions such as exponential and lognormal distributions, using the *poweRlaw* R package available at https://github.com/csgillespie/poweRlaw. Each model was fitted using a cut-off value, *x*_min_, which is estimated by minimizing the Kolmogorov–Smirnoff (KS) statistic. To assess whether one or more of these distributions were a good fit or not, each of these distributions was fitted with and without *x*_min_ values [42]. If either the exponential distribution or lognormal distribution is a good fit to degree distribution of the gene flow network, then this could imply that power-law distribution may not be a good fit to the data. If both the exponential and lognormal distributions are not good fits, whereas the power law is, then scale-free and other associated properties could be governing the gene flow network as well, just like numerous networks in many different fields [43,44].

### 2.7. Interaction coefficient

The interaction coefficient (IC) represents the degree of interaction (specific to type of interaction, e.g. native–alien or alien–alien) between two taxa, obtained as the number of interactions of a specific type normalized by total number of interactions for a given pair of taxa. Let us consider two taxa, *D*1 and *D*2, in the gene flow network. Different types of interactions between gene clusters in *D*1 and *D*2 are (a) genes from the native cluster(s) of *D*1 transferred to *D*2 and is recovered as alien cluster(s) in *D*2 (*N_D_*_1_ → *A_D_*_2_), (b) *D*1 receiving genes from the native cluster(s) of *D*2 (*A_D_*_1_ ← *N_D_*_2_), and (c) exchange of genes between alien genome components of both taxa (*A_D_*_1_ ↔ *A_D_*_2_). For a given pair *D*1 and *D*2, the IC for each possible type of interaction is calculated as follows:

$$\begin{array}{rl} & \mathrm{IC}(N_{D1}\to A_{D2})\\ & \;=\;\left(\begin{array}{c} \mathrm{Number}\;\mathrm{of}\;\mathrm{interactions}\;\mathrm{of}\;\mathrm{type}\;N_{D1}\;\\ \to A_{D2}\;(\mathrm{transfer}\;\mathrm{of}\;\mathrm{genes}\;\mathrm{from}\;\mathrm{native}\;\mathrm{cluster}(s)\;\\ \;\mathrm{of}\;D1\;\mathrm{to}\;D2)\; \end{array}\right)/\mathrm{TI}, \end{array}$$

$$\begin{array}{rl} & \mathrm{IC}(A_{D1}\leftarrow N_{D2})\\ & \;=\;\left(\begin{array}{c} \mathrm{Number}\;\mathrm{of}\;\mathrm{interactions}\;\mathrm{of}\;\mathrm{type}\;A_{D1}\;\\ \leftarrow N_{D2}\;(\;D1\;\mathrm{receiving}\;\mathrm{genes}\;\mathrm{from}\;\mathrm{native}\;\;\\ \;\mathrm{cluster}(s)\;\;\mathrm{of}\;D2)\; \end{array}\right)/\mathrm{TI} \end{array}$$

$$\begin{array}{rl} & \mathrm{and}\;\mathrm{IC}(A_{D1}↔A_{D2})\\ & \;=\left(\begin{array}{c} \mathrm{Number}\;\mathrm{of}\;\mathrm{interactions}\;\mathrm{of}\;\mathrm{type}\;A_{D1}\;\\ ↔A_{D2}(\mathrm{exchange}\;\mathrm{of}\;\mathrm{genes}\;\mathrm{between}\;\mathrm{alien}\;\;\\ \;\mathrm{components}\;\;\mathrm{of}\;D1\;\mathrm{and}\;\;D2)\; \end{array}\right)\;/TI, \end{array}$$

where TI denotes total interactions (total number of connections between *D*1 and *D*2).

This is calculated for all pairs of taxa and here we performed this analysis at the phylum level; however, this analysis can also be performed at other taxonomic levels. Pairwise IC for each interaction type for each phylum pair enables assessment of favourability of mode of transfer (native–alien or alien–alien) between two taxa under considerations. Note that connections between native clusters of two different organisms may also arise in the network, however, this could be between organisms with native genomes of high compositional similarity, e.g. strains of a species, and may not be indicative of horizontal transfer (transfers among conspecific strains via homologous recombination were ignored), and are therefore not considered here (a total of 1389 such connections (approx. 2%) were detected but excluded from the horizontal gene flow network).

### 2.8. Functional classification of genes

We performed the functional classification for each alien cluster using eggNOG-mapper [45]. The entire COG database was downloaded, and command-line eggNOG-mapper was run for each alien cluster.

## 3. Results

### 3.1. Gene-clustering-based network

A semi-directed network of putative HGT was constructed following annotation of clusters generated by the Jenson-Shannon Codon Bias (JS-CB) approach as native and alien. First, genes with similar codon usage bias were grouped together for each genome using an agglomerative clustering procedure implemented in JS-CB (see Methods). For each genome, the largest cluster, typically comprised over 60% of the genes, was identified as native, and the smaller clusters of atypical composition were subjected to marker gene enrichment and phyletic pattern analyses. The atypical clusters that have enrichment of marker genes often associated with horizontal acquisition or have a majority of genes displaying aberrant phyletic pattern were annotated alien, and the remaining atypical clusters were annotated as native.

Application of JS-CB to 708 prokaryotic genomes resulted in a total of 6323 clusters. Marker enrichment analysis was performed on atypical clusters generated by JS-CB to identify clusters enriched in marker genes that are typically associated with HGT (see Methods). As a sanity check, we also performed marker enrichment analysis on largest (typical or native) gene cluster for each genome; here, the fold enrichment of markers was obtained relative to the whole genome background. These native clusters were not expected to be enriched. The fold-enrichment values for the native clusters were found to range from 0.47 to 1.3. We used this as a basis to establish 1.25 as the enrichment cut-off (only two genomes had native clusters with fold-enrichment over 1.25 (genome G261: *Marinobacter aquaeolei* VT8 and genome G393: *Pyrobaculum calidifontis* JCM 11548 in electronic supplementary material, table S2). For each atypical gene cluster in a genome, fold-enrichment of markers was obtained relative to the native cluster of the genome. In total, 2090 (approx. 38% of atypical clusters generated by JS-CB) atypical clusters across 708 genomes were found to be enriched in marker genes. A few marker genes were found in 1893 (approx. 29% of all clusters generated by JS-CB) atypical clusters but were not found to be enriched. No marker gene was found in 1652 (approx. 26% of all clusters generated by JS-CB) atypical clusters (electronic supplementary material, table S2). The atypical clusters enriched in marker genes were annotated alien. Being not enriched does not necessarily mean not alien, as the marker genes may have been lost with the passage of time after acquisition. We therefore subjected the non-enriched clusters to phyletic pattern analysis to identify clusters displaying unusual phyletic pattern and thus indicative of horizontal acquisition.

**Figure 1.**
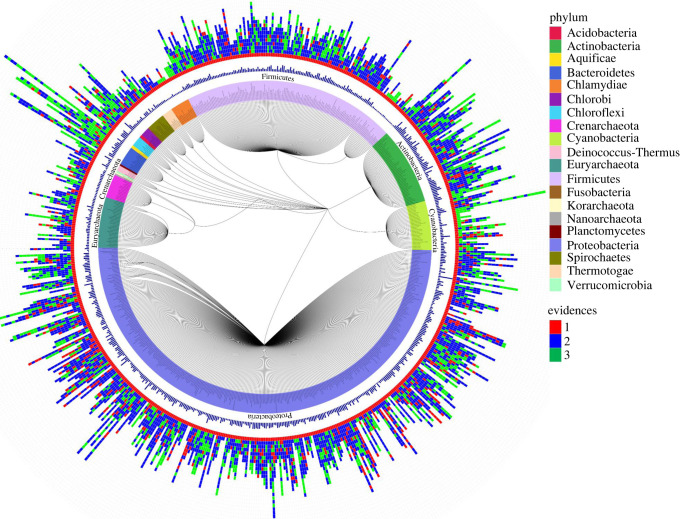
Annotation of native and alien clusters. Phylogenetic tree (unrooted) of approx. 700 species representing the taxonomic relationship generated using NCBI Taxonomy Browser. The first lane from inside represents the species name, colour-coded as the phylum it belongs to. The second lane is a bar-plot of genome size of all the genomes. The next lane is the representation of all clusters detected for each of the genomes (each rectangular box is one cluster). A detailed account of each cluster has been provided as electronic supplementary material, tables S3–S4. Evidence 1 indicates only compositional atypicality; evidence 2 indicates both compositional atypicality and marker enrichment, or compositional atypicality and unusual phyletic pattern; and evidence 3 indicates compositional atypicality, marker enrichment and unusual phyletic pattern.

APP was applied to the non-enriched atypical clusters to examine phyletic pattern of genes harboured by the atypical clusters. If a majority of the genes in an atypical cluster displays aberrant phyletic pattern, i.e. absent or present in only few close relatives of the genome harbouring the genes (see Methods), the atypical cluster was annotated alien. Thirty-eight of 708 genomes lacked close relatives with completely sequenced genomes (none of the known relatives within the same species, genus, or family rank have their genomes completely sequenced). Therefore, for these genomes, phyletic pattern analysis was not performed. Of the total of 3545 non-enriched atypical clusters, 1769 have a majority of their genes displaying aberrant phyletic pattern and were, therefore, annotated alien. The remaining atypical clusters (1571, 27%) were annotated native (figure 1; electronic supplementary material, tables S3–S4). We thus obtained a total of 2486 native clusters (approx. 40%) and a total of 3837 alien clusters (approx. 60%), which were used in constructing the gene flow network as described below.

**Figure 2.**
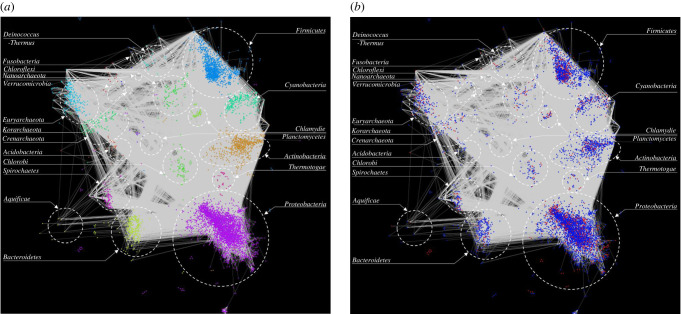
HGT network. A gene-clustering-based network was computed based on Jenson–Shannon codon usage Bias. Genes with similar codon usage bias were grouped together to form clusters. Pairs of similar clusters (nodes) in different genomes were identified and plotted using Cytoscape. (*a*) Network shows all inter-phylum connections in grey. Each phylum is colour coded and labelled. (*b*) Inter-phylum connections (gene exchange) along with native (red) and alien (blue) cluster designations.

Following annotation of JS-CB clusters, pairwise inter-genome cluster comparisons were performed within the same hypothetical testing framework. Pairs of similar clusters in different genomes were identified and then a network with nodes denoting clusters and edges connecting nodes signifying similarity of the nodes was constructed and plotted using Cytoscape (figure 2). Connections (edges/links) between native and alien clusters in the network indicate transfer of native genes from one genome to another, whereas the connections between alien clusters indicate transfer of alien genes from one genome to another. In figure 2*a*, we show the inter-phylum connections (gene exchange across phyla) along with native and alien cluster designations. Each node (donor/recipient) is colour coded according to their host phylum (figure 2*a*). Figure 2*b* shows the same network but with cluster type (native/alien) indicated. The majority of the nodes in the network are of alien origin (shown in blue in figure 2*b*).

**Figure 3.**
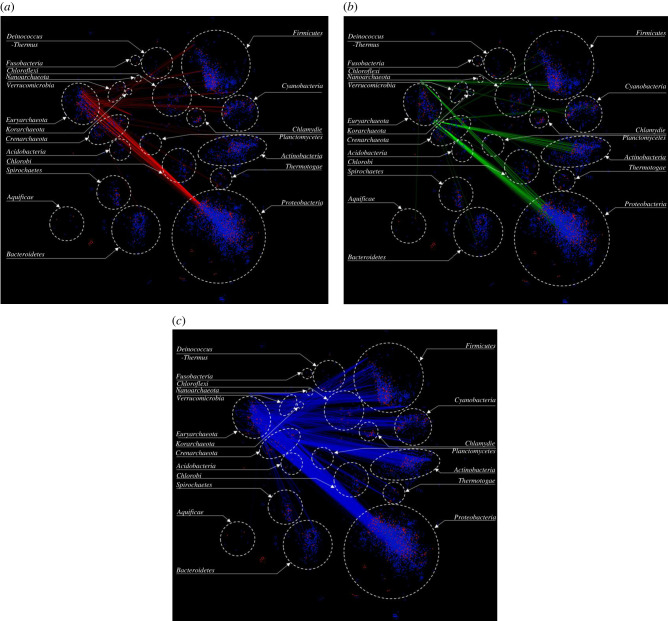
Gene clustering network showing links of Euryarchaeota. (*a*) All links shown in red represent gene transfers from genomes belonging to phylum Euryarchaeota (donors) to other phyla(recipients). (*b*) All links shown in green represent gene acquisitions into the genomes of Euryarchaeota (recipients) from native components of genomes from other phyla(donors). (*c*) All links shown in blue represent gene mobilization between alien components of genomes of Euryarchaeota and alien components of genomes of other phyla.

For a given phylum, the links between clusters from this phylum and those from other phyla in the network refer to: (1) transfer of native genes from this phylum to other phyla (native–alien cluster pair in this order, with transfer direction from the given phylum to all other phyla), (2) acquisition of genes in this phylum from native components of genomes belonging to other phyla (alien–native cluster pair in this order, with transfer to the given phylum from other phyla), and (3) transfer of alien genes in this phylum to other phyla or acquisition in this phylum of genes harboured by alien clusters of other phyla (alien–alien cluster pair with direction of transfer indeterminate). As an example, we show in figure 3*a–c* all connections for Euryarchaeota. Figure 3*a* displays transfers of native genes from organisms belonging to Euryarchaeota to other phyla including Firmicutes, Cyanobacteria, Actinobacteria, Thermotogae and Proteobacteria, while figure 3*b* shows transfers of native genes from other phyla, including Firmicutes, Cyanobacteria, Actinobacteria, Thermotogae, Proteobacteria, Bacteroidates, Aquificae, Chlorobi and Verrucomicrobia, to Euryarchaeota. A relatively higher influx of genes was observed from the phyla Actinobacteria and Proteobacteria (figure 3*b*). Figure 3*c* displays transfer of already resident alien genes of Euryarchaeota to other phyla and of alien genes in other phyla to Euryarchaeota.

**Figure 4.**
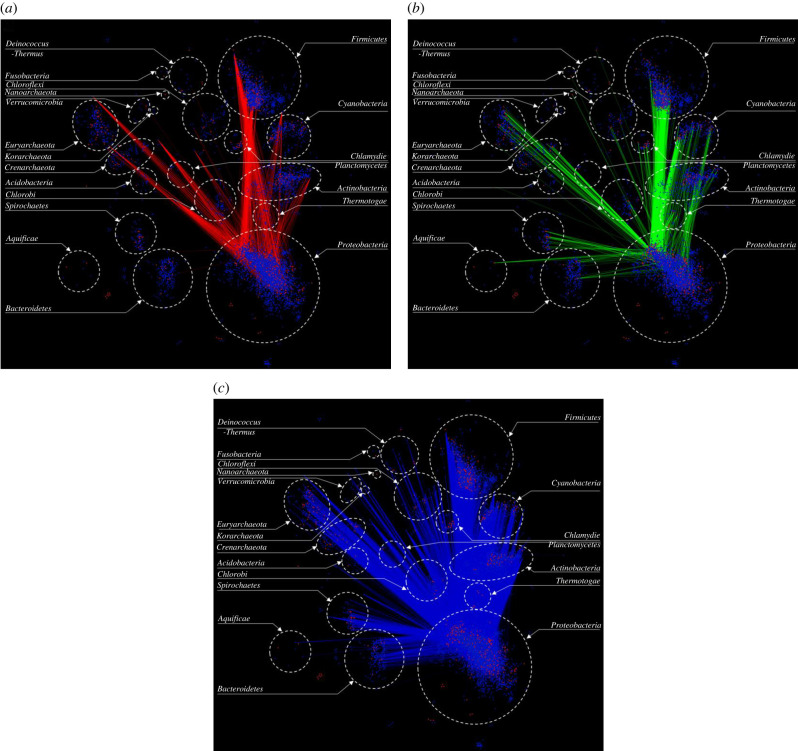
Gene clustering network showing links of Proteobacteria. (*a*) All links shown in red represent gene transfers from genomes belonging to phylum Proteobacteria (donors) to other phyla (recipients). (*b*) All links shown in green represent gene acquisitions into the genomes of Proteobacteria (recipients) from native components of genomes from other phyla (donors). (*c*) All links shown in blue represent gene mobilization between alien components of genomes of Proteobacteria and alien components of genomes of other phyla.

Among the phyla represented by over 700 sampled genomes, Proteobacteria has the largest number of clusters associated with gene exchange; in figure 4*a–c*, we show the network for Proteobacteria. Manifold instances of gene transfer from the native components of Proteobacterial genomes to Firmicutes were observed (figure 4*a*). This is consistent with several previous studies that have reported numerous instances of HGT between Firmicutes and Proteobacteria [46,47]. As mentioned previously, a considerable number of proteobacterial genes are inferred to have been acquired by Euryarchaeota. Other phyla, such as Actinobacteria, Cyanobacteria, and Crenarchaeota, also appear to have acquired many genes from Proteobacteria (figure 4*a*). In figure 4*b*, we show the network for transfer of genes from the native components of genomes from other phyla to the genomes of Proteobacteria. Coincidentally, among all phyla, Firmicutes have mobilized the highest number of their native genes to Proteobacteria, suggesting high degree of interactions (gene exchange) between organisms from these phyla. Figure 4*c* shows links between alien clusters of Proteobacteria and those of other phyla.

### 3.2. Gene flow network is scale-free

The total number of edges (interactions) in the gene flow network was found to be over approximately 98 000. The degree distribution of the network was observed to be better fitted by power-law than the other heavy tail distributions [42]. By the application of Network Analyzer feature of Cytoscape, we extracted the node-degree distribution of the network (electronic supplementary material, figure S1). The parameters calculated for the power-law distribution were *x*_min_ = 101 and *α* = 2.253445 (see Methods for the description of the parameters). Of 2500 KS tests performed on the network, 2490 (approx. 99.6%) failed to reject the null hypothesis that the data were from power-law distribution. We further observed that all the KS tests rejected the null hypothesis that the data were generated from an exponential distribution (estimated parameters for exponential distribution with *x*_min_: *λ* = 0.001622268 and *x*_min_ = 512). For exponential distribution without *x*_min_, we obtained a *λ* = 0.03154196. Similar to the exponential with *x*_min_, all of the KS tests rejected the null hypothesis that the distribution is exponential. We also observed that all the KS tests rejected the null hypothesis that the distribution is lognormal (for lognormal distribution with and without *x*_min_, the parameter values were *µ* = 2.602942 and *σ*^2^ = 1.291843). Overall, since the exponential and lognormal distributions, with or without the *x*_min_ parameter, were not good fits to the data and only the power law fitted well, this led us to infer that the gene flow network is scale-free. Also, as shown in electronic supplementary material, figure S1, we observed that a vast number of nodes of the HGT network has a small degree but many of them are connected to a few hub nodes with very large degrees. Thus, the degree of a randomly selected node would likely be tiny or arbitrarily large, indicating that the gene flow network lacks internal scale [48]. Our analysis thus supports scale-free structure of the network.

### 3.3. Inter-phylum interaction analysis reveals preferred mode of transfer

In the gene flow network, the interaction (connection) between clusters from two representative phyla is assessed in terms of interaction coefficient (IC; see Methods). For determining the preferred mode of transfer, we analysed the IC values for all modes (*N_D_*_1_ → *A_D_*_2_, *A_D_*_1_ ← *N_D_*_2_ and *A_D_*_1_ ↔ *A_D_*_2_) for each phylum pair; the higher the value for a mode, the more the preference to mobilize genes by that mode of transfer.

**Figure 5.**
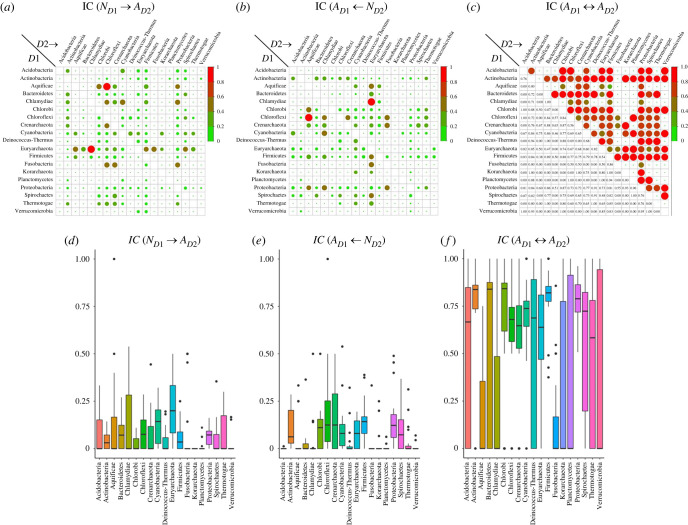
Inter-phylum interaction analysis. The degree of interaction between two gene sharing clusters are represented in terms of interaction coefficient (IC; see methods). (*a-b*) Plots of the IC(*N_D_*_1_ → *A_D_*_2_), IC(*A_D_*_1_ ← *N_D_*_2_) for all phylum pairs considering donation and acquisition events occurring between native–alien cluster pairs. (*c*) Plot of the IC(*A_D_*_1_ ↔ *A_D_*_2_) values for all phylum pairs considering shared genes belonging to alien clusters. Phylum-wise distribution plot of (*d–f*) IC(*N_D_*_1_ → *A_D_*_2_), IC(*A_D_*_1_ ← *N_D_*_2_) and IC(*A_D_*_1_ ↔ *A_D_*_2_).

The preferential modes of transfer between any two phyla can be gleaned from figure 5*a–c*. To understand the preferences of modes of transfer among different phyla, we focused our attention on phylum pairs. Here, as examples, we first consider interactions (connections) between Chloroflexi and other phyla, and then we consider interactions between Proteobacteria and other phyla. For inter-phylum transfers, Chloroflexi–Aquificae pair has the highest IC value of 1 for transfer of native genes from Aquificae to Chloroflexi (figure 5*a*; electronic supplementary material, table S5). This means that bacteria belonging to Aquificae donated their native genes to the genomes of Chloroflexi but not the other way round, and also no alien–alien exchange was observed. The IC(*N_D_*_1_ → *A_D_*_2_) value of 0.5 for Chloroflexi (*D*1) and Fusobacteria (*D*2) pair (electronic supplementary material, table S5) indicates that of all interactions between genomes of these two phyla, half of them involve native genes of Chloroflexi being donated to genomes belonging to Fusobacteria. The other half was involved in alien-to-alien mode of transfer with IC(*A_D_*_1_ ↔ *A_D_*_2_) value of 0.5. We also observed incidences of gene transfer between Chloroflexi and multiple phyla belonging to Archaea. For example, the IC(*N_D_*_1_ → *A_D_*_2_) value for Chloroflexi–Crenarchaeota pair was found to be 0.44. We also observed other inter-domain gene transfer events, such as, native genes from Euryarchaeota transferred to Chlamydiae; the IC(*N_D_*_1_ → *A_D_*_2_) value for this was found to be 1, implying that this is the only detectable mode of transfer between these two phyla. For the phylum pair Chloroflexi (*D*1) and Crenarchaeota (*D*2), IC(*N_D_*_1_ → *A_D_*_2_) = 0.45 and IC(*A_D_*_1_ ↔ *A_D_*_2_) = 0.55, indicating transfer of native genes from Chloroflexi to Crenarchaeota and not the other way round, and with almost similar frequency, exchange of genes between the alien components of genomes from these taxa. Differential values of IC(*N_D_*_1_ → *A_D_*_2_), IC(*A_D_*_1_ ← *N_D_*_2_) and IC(*A_D_*_1_ ↔ *A_D_*_2_) for a phylum pair involving Chloroflexi (*D*1) reveals a bias towards a particular mode of transfer of genes depending on the other phylum (*D*2) involved (figure 5*a–c*; electronic supplementary material, tables S5 and S6).

Next, we explored the interactions between genomes belonging to Proteobacteria (*D*1) and other phyla (*D*2). The tendency to prefer a particular mode of transfer was again observed and was represented in the form of differential IC values. Interactions with Chlamydiae entailed abundance of transfers of native genes from Proteobacteria (IC(*N_D_*_1_ → *A_D_*_2_) = 0.49). The lowest value for IC(*N_D_*_1_ → *A_D_*_2_) was observed with Planctomycetes (0.04), indicating that very few native genes were transferred from Proteobacteria to Chlamydiae. Here also we observed multiple inter-domain transfer events, between proteobacterial and archaeal genomes. For example, Proteobacteria donated native genes to Crenarchaeota (IC(*N_D_*_1_ → *A_D_*_2_) = 0.28). A similar range of IC values for transfer of proteobacterial native genes was found for several other recipient phyla groups, such as Chloroflexi, Euryarchaeota and Spirochaetes (IC(*N_D_*_1_ → *A_D_*_2_) = 0.27–0.24). For transfer events involving proteobacteria and archaea, a preference towards alien-to-alien mode of transfer was observed in general (IC(*A_D_*_1_ ↔ *A_D_*_2_) values ranging from 0.7 to 0.93). For some phyla such as Planctomycetes and Verrucomicrobia, almost all of the gene exchange with Proteobacteria was found to be of alien-to-alien mode (IC(*A_D_*_1_ ↔ *A_D_*_2_) values ranging from 0.95–0.97).

For a given pair of phyla, we further analysed the IC(*A_D_*_1_ ↔ *A_D_*_2_) values that signify the flow of mobilome (alien genes) across phyla, along with the IC(*N_D_*_1_ → *A_D_*_2_) and IC(*A_D_*_1_ ← *N_D_*_2_) values signifying horizontal flow of native genes, for each phylum pair (*D*1 and *D*2) (figure 5*c,f*; electronic supplementary material, table S6). Gene transfer between certain phyla, such as phylum pairs Acidobacteria–Chlorobi, Euarychaeota–Planctomycetes, etc., occurs via alien-to-alien mode only (IC(*A_D_*_1_ ↔ *A_D_*_2_) = 1). We further observed for phylum pairs involving Fusobacteria, Korarchaeota, Planctomycetes and Verrucomicrobia, the distribution of the IC(*A_D_*_1_ ↔ *A_D_*_2_) values are dispersed (positively skewed) mostly over the interquartile range (figure 5*f*) with a wide dispersion. This suggests that for these phyla, the preferential mode of transfer is alien-to-alien.

Alien-to-alien links between genomes from different phyla might represent singular or multiple transfer events. By accounting for number of interactions between two phyla and quantifying them in terms of interaction coefficients (IC), preference towards a particular mode of transfer was revealed. Previous BLAST-based networks have detected many potential transfer events, but haven't shed a light on the aspect of mode of transfer between taxa. The gene exchange network presented here reveals that the preferred mode of transfer is between alien components of the genomes (figure 5). While native–alien cluster designations aided in determining the direction of transfer events, abundance of alien-to-alien connections suggest the existence of a shared mobilome and from which genes are exchanged frequently. Alien-to-alien connections representing the shared ‘mobilome’ can be extracted from primary network file ‘alien-to-alien_mobilome.txt’ in the GitHub repository at https://github.com/sohamsg90/Gene-flow-network.

Notably, there were many instances of preference for native-to-alien mode of transfer. However, we observed an overall prevalence and dominance of alien-to-alien mode of transfer in the network. Though not unexpected, this is an important finding reinforcing the existence of a shared mobilome facilitating frequent horizontal gene flow among prokaryotes.

### 3.4. Differential gene flow

The gene clustering-based network enabled visualization of gene flow from one taxon to another. We then performed quantification by normalizing the number of links between two taxa by all possible inter-taxon genome pairs for the taxon pair, that is, by dividing the number of observed links between two taxa by the number of all possible inter-taxon genome pairs for the taxon pair. This is provided, as number of links per 100 inter-phylum genome pairs, for each phylum pair in electronic supplementary material, table S7.

In addition to inter-phylum transfers, we also quantified the intra-phylum transfers. Among the intra-phylum transfers, the highest number of links per 100 genome pairs was observed within the phylum Chlorobi (approx. 53 links per 100 genome pairs). This was closely followed by Bacteroidetes and Actinobacteria with approximately 52 and approximately 51 links per 100 genome pairs, respectively. For Archaea, the most abundant intra-phyla transfers were observed within Crenarchaeota and Euryarchaeota (approx. 9 links per 100 genome pairs). Considering inter-phylum transfer events, and following the exclusion of the Acidobacteria–Verrucomicrobia pair that has only two genomes from Acidobacteria and one genome from Verrucomicrobia and three links between them, the Actinobacteria–Verrucomicrobia pair has the highest number of links following normalization, approximately 55 links per 100 genome pairs. This is followed by the Actinobacteria–Chlorobi pair, with approximately 40 links per 100 genome pairs, and then the Deinococcus-Thermus-Chlorobi pair, with approximately 37 links per 100 genome pairs. This suggests that genomes within these phylum pairs are relatively more interactive, in terms of gene exchange across phyla, within the gene flow network. We caution that a link signifies a connection between native and alien gene clusters or alien and alien gene clusters, across two phyla under consideration, and it is, therefore, plausible that a link may be representing multiple transfer events between the phyla, however, it does not indicate number of horizontal transfer events. We also observed multiple links between phyla belonging to Archaea and Bacteria, where Chlorobi-Euryarchaeota links were more abundant than other archaea–bacteria pairs, with 21 links per 100 genome pairs.

Overall, intra-bacteria and intra-archaea linkages are most dominant, much more than inter-domain (bacteria–archaea) linkages, as expected (electronic supplementary material, table S7). This is also reflected in general for intra-phylum linkages compared with inter-phylum linkages.

### 3.5. Functional classification of horizontally acquired genes

To perform functional classification of horizontally acquired genes, we employed the eggNOG mapper for each linked alien cluster in the network. For each phylum, the COG categories for all alien genes were recorded. Considering all alien genes from different phyla, genes involved in metabolism were found most numerous (*n* = 663 948; approx. 39%; table 1), followed by genes associated with information storage and processing (*n* = 371 743; approx. 22%; table 1), genes of uncharacterized functions (*n* = 361 017; approx. 21%; table 1), and genes involved in cellular processing and signalling (*n* = 301 761; approx. 17%; table 1). We observed that the number of mobile genes associated with metabolism is almost double of those from other COG categories (table 1).

**Table 1.** Functional enrichment of shared genes. The detailed account of COG-types, their functional description, count, percentage and summation under general categories are provided.

COG general category	COG-type	count	sum	COG functional description
information storage and processing	A	4409 (0.26%)	371 743 (21.89%)	RNA processing and modification
	B	14 483 (0.85%)		chromatin structure and dynamics
	J	95 917 (5.65%)		translation, ribosomal structure and biogenesis
	K	131 694 (7.75%)		transcription
	L	125 240 (7.37%)		replication, recombination and repair
cellular processes and signalling	D	33 929 (2%)	301 761 (17.77%)	cell cycle control, cell division, chromosome partitioning
	M	96 705 (5.69%)		cell wall/membrane/envelope biogenesis
	N	41 887 (2.47%)		cell motility
	O	54 694 (3.22%)		post-translational modification, protein turnover and chaperones
	T	46 600 (2.74%)		signal transduction mechanisms
	U	26 964 (1.59%)		intracellular trafficking, secretion and vesicular transport
	V	538 (0.03%)		defence mechanisms
	W	420 (0.02%)		extracellular structures
	Y	24 (approx. 0%)		nuclear structure
metabolism	C	99 325 (5.85%)	663 948 (39.09%)	energy production and conversion
	E	114 130 (6.72%)		amino acid transport and metabolism
	F	58 491 (3.44%)		nucleotide transport and metabolism
	G	83 284 (4.9%)		carbohydrate transport and metabolism
	H	65 761 (3.87%)		coenzyme transport and metabolism
	I	63 373 (3.73%)		lipid transport and metabolism
	P	98 157 (5.78%)		inorganic ion transport and metabolism
	Q	81 427 (4.79%)		secondary metabolites biosynthesis, transport and catabolism
uncharacterized	R	291 923 (17.19%)	361 017 (21.26%)	general function prediction only
	S	69 094 (4.07%)		function unknown

In addition to the aforementioned broad classification, we analysed mobile genes for their membership in specific COG classes. The highest count was found to be for class R, which represents general function prediction only (*n* = 291 923; approx. 17%; table 1). This was followed by K (transcription; *n* = 131 694; approx. 7.75%; table 1), L (replication, recombination and repair; *n* = 125 240; approx. 7.37%; table 1), and E (amino acid transport and metabolism; *n* = 114 130; approx. 6.7%; table 1). Next, we observed that genes with functions related to energy production (C; *n* = 99 325; approx. 5.8%; table 1), ion transport (P; *n* = 98 157; approx. 5.7%; table 1), membrane biogenesis (M; *n* = 96 705; approx. 5.6%; table 1), and translation (J; *n* = 95 917; approx. 5.6%; table 1) were enriched. Genes involved in defence mechanisms (V; *n* = 538; approx. 0.03%; table 1) were also found to be mobilized. Among all the COG classes, genes associated with nuclear structure (Y; *n* = 24; approx. 0.001%; table 1) and extracellular structures (W; *n* = 420; approx. 0.02%; table 1) were least numerous.

Functional analysis revealed a substantial number of information processing genes to have been mobilized. Previous studies have reported genes associated with metabolic functions to be exchanged more frequently than the information processing genes (including replication, transcription and translation) [29,49–54]. The lack of mobility of information processing genes has been attributed to their roles within complex structures [53]. Proteins that function in a complex structure, e.g. ribosomal proteins, are adapted to their common function. An HGT event that results in the replacement of such a gene with a less adapted homologue will result in reduced fitness of the recipient [53]. Recently, Cohen *et al.* [55] tested the relative impact of functional category and the number of interacting partners on HGT frequency. Their result showed that the complexity hypothesis still passes a reality test in the genomic era. However, HGT barriers owing to multiple interacting partners are not restricted to information processing genes only, but may be observed in all functional categories [55]. Acquisition frequency of metabolic genes depends on their role within the cellular metabolic network [56]. A study of the laterally acquired genes within the *E. coli* metabolic network showed that HGT is more frequent among enzymes involved in peripheral reactions (uptake and metabolism of nutrients) in comparison to those involved in central reactions (biomass production) [56]. Popa *et al.* [54], in their study to find genomic barriers to lateral gene transfer, found that information processing genes accounted for over approximately 11% of the transferred events. A similar result was reported by Garcia-Vallve *et al.* [49], based on compositional features in bacterial and archaeal genomes. They observed that even though metabolic genes account for highest number of genes exchanged, there were multiple instances of informational genes being exchanged (1.1–19.1%) across bacterial lineages. This trend is also observed in our network, where the highest number of genes were associated with metabolism, closely followed by information processing genes.

### 3.6. Functional annotation of alien genes shared across major phyla

**Figure 6.**
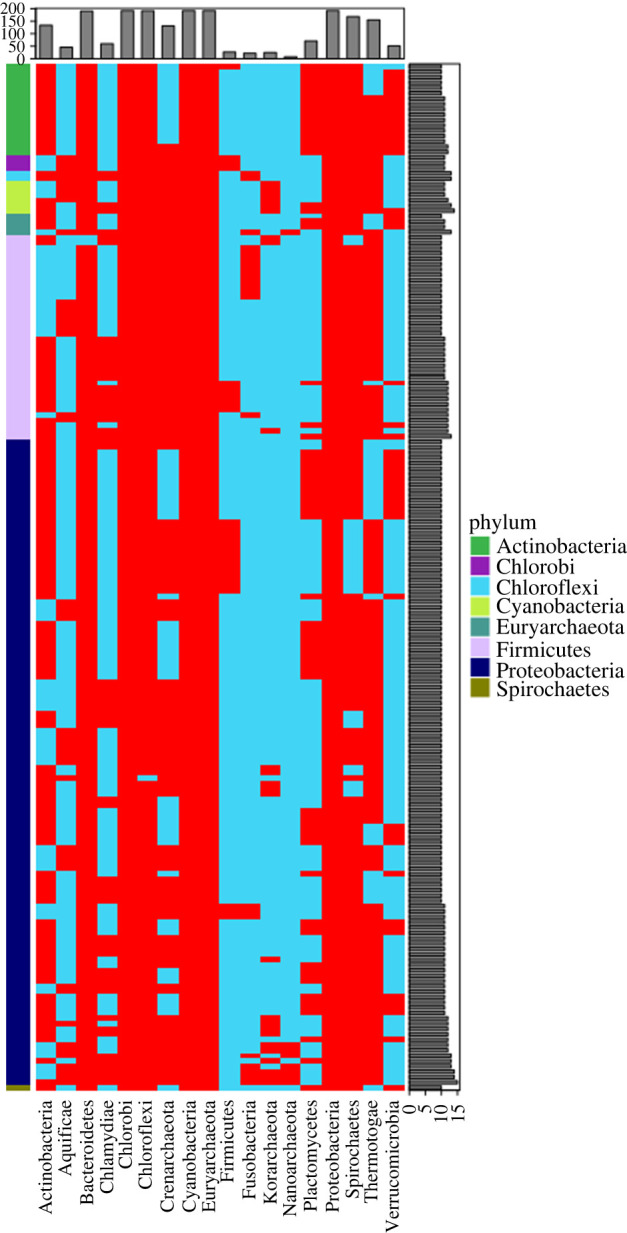
Detection of over-enriched gene sets. Genes that are overrepresented (*n* = 192) across the network are represented in the form of a heatmap. Each row is a gene, the presence (red) or the absence (blue) of which are marked for each phylum under consideration. The occurrence of a particular gene across all different phyla are provided as a bar-plot on the right side. The total number of genes out of 192 is summed to be represented as the bar-plot on the top. A detailed account of the analysis has been provided as electronic supplementary material, table S8.

We further examined alien genes representing different functional categories that were found across different phyla. Of all alien genes that appeared more than once, we selected genes that were present in more than 10 phyla. Figure 6 shows the occurrence of 192 genes representing different functional categories that were shared across many different phyla. These genes belong to the phyla Actinobacteria (*n* = 17), Chlorobi (*n* = 3), Chloroflexi (*n* = 2), Cyanobacteria (*n* = 6), Euryarchaeota (*n* = 4), Firmicutes (*n* = 38), Proteobacteria (*n* = 121) and Spirochaetes (*n* = 1). Based on the functional annotation, we identified these genes to be encoding proteins that are phage-related, membrane-fusion, immunity-associated, involved in protein-protein interaction or classified as transposase, integrase, recombinase. For example, one of the genes, belonging to *Clavibacter michiganensis*, was found to encode putative AbiEII toxin belonging to Type IV toxin–antitoxin (TA) system. Bacteria resist phage infection using multiple strategies, including CRISPR-Cas and abortive infection (Abi) systems. Abi systems provide population-level protection from phage predation, via ‘altruistic’ cell suicide. It has recently been shown that some Abi systems function via a toxin–antitoxin mechanism rendered by the widespread AbiE family. The *Streptococcus agalactiae* AbiE system consists of a bicistronic operon encoding the AbiEi antitoxin and AbiEii toxin, which function as a Type IV toxin–antitoxin system [57].

Next, we also observed a phage tail tape measure protein to be frequently shared among different phyla. Phages play critical roles in the spread of virulence factors [58] and extended-spectrum β-lactamase and fluoroquinolone resistance genes [59]. An essential step in the phage life cycle is genome entry, where the infecting phage must productively interact with the components of the bacterial cell envelope to transmit its genome out of the viral particle and into the host cell cytoplasm. The phage tail tape measure protein is an inner membrane protein, and is found to act as a periplasmic chaperone in the genome injection process [60].

We also found an AIPR protein, a part of Abi system, shared across many phyla. This protein consists of two domains: PoNe (Polymorphic Nuclease Effector) and AIPR (Abortive Infection Phage Resistance). PoNe belongs to a diverse superfamily of PD-(D/E)xK phosphodiesterases, and is associated with several toxin delivery systems including type V, type VI and type VII [61]. AIPR family proteins are suggested to be often associated with restriction modification system [62,63].

We also identified multiple phage integrase proteins that are known to mediate unidirectional site-specific recombination between two DNA recognition sequences, the phage attachment site, *attP* and the bacterial attachment site, *attB* [64]. We also found multiple instances of the well-known transposases associated with OrfAB family. Genes encoding these transposases are known to be components of mobile genetic elements of bacterial genomes. Gene encoding toxin HlyA of the type I secretion system (T1SS) was also found to be frequently shared. The T1SS is composed of an inner membrane ABC transporter HlyB, an outer-membrane channel protein TolC and a membrane fusion protein HlyD. Toxin HlyA of *Escherichia coli* has been reported to be exported without a periplasmic intermediate by the T1SS [65]. We also observed sharing of Penicillin binding protein and small multidrug resistance protein-encoding genes. These have been known to be frequently mobilized [8,27,38,66]. We also identified several genes of unknown functions that are frequently shared. Complete details have been provided in electronic supplementary material, table S8.

### 3.7. Cluster-wise analysis of the node with the highest degree

The node-degree distribution plot, as shown in electronic supplementary material, figure S1, depicts the frequency of nodes (clusters) as a function of degree (number of connections to a node). As previously discussed, we observed a large number of nodes with smaller degrees and a few nodes with much higher degrees, that is, the hub nodes. G463_Cl_3 node (cluster) was found to have the largest number of connections in the network. This cluster was annotated as alien and belongs to *Staphylococcus aureus* subsp. *aureus* NCTC 8325 of phylum Firmicutes. Over 2500 of 3225 connections (approx. 78%) were recognized as signifying acquisitions of genes from alien components of non-Firmicute genomes (electronic supplementary material, figure S2). This node is connected to numerous alien clusters across phyla. Gene transfers of the alien-to-alien transfer mode (alien clusters marked in blue in electronic supplementary material, figure S2) from other nodes connected with this node are quite plausible, with this node acting as a central node or one of the connecting nodes. Indeed, we observed that these alien clusters are connected to alien clusters of other genomes across phyla, revealing flow of alien genes in a shared mobilome. We further analysed the putative functions of the genes that constitute this cluster in *S. aureus* subsp. *aureus* NCTC 8325 using the eggNOG-mapper. We could only identify the function of one gene, annotated as a transposase. Transposase has been known to be associated with mobile genomic elements [8,27,66].

## 4. Discussion

The gene-clustering-based network presented here shines a new light on HGT events that have driven the evolution of prokaryotes. A limitation of BLAST-based approaches is their inability to infer the direction of gene transfer, i.e. the identification of donor and recipient of a mobilized gene. The JS-CB based network presented here addresses this by first generating gene clusters representing potentially distinct sources, annotating the clusters as native and alien aided by marker enrichment and phyletic pattern analysis, and then performing inter-genome clustering. The final pass native and alien clusters are supported by multiple lines of evidence that place a high confidence over their annotation as alien or native. The mobilization of alien genes across taxa and the donor and recipient identities are established within the same statistical hypothesis testing framework of JS-CB, resulting in a gene exchange network that enables visualization of putative transfer events among prokaryotes.

The gene exchange network reveals interactions among prokaryotes from phylogenetically close and distant taxa. Although phylogenetically proximal organisms with similar genome composition are expected to more readily exchange and integrate genetic materials, a plethora of transfers among phylogenetically distant organisms (e.g. those from different phyla) suggests a selection pressure on genes enabling their cross-over across the phylogenetic barrier and integration and maintenance in new host genomes.

Phylogenetic methods are known to be more sensitive to reconstructing ancient transfer events; however, note that most of the transferred genes do not confer long-term benefits and are eventually lost from the recipient genomes. Thus, at a snapshot of time, most of the resident alien genes in a genome are likely recently acquired genes, which can be detected and quantified using composition-based methods such as JS-CB that exploits codon usage bias to segregate genes acquired from different sources. Furthermore, to identify a high confidence set of alien genes, we considered only those compositionally atypical clusters as of foreign origin that were also supported by phyletic pattern or marker enrichment-based analysis. The phyletic pattern analysis was performed using our just developed comparative genomics tool APP whose accuracy, however, depends on how enriched the database is. Our set of over 700 representative completely sequenced genomes has often several close relatives of query genomes available to perform a robust comparative analysis at different taxonomic levels (species, genus and family levels).

Following annotation of clusters as native and alien for each genome, inter-genome clustering was performed that shed a light on the mobilization of genes from the native component of one genome to another genome where they were recovered as an alien cluster and from alien component of one genome to another where they were recovered as an alien cluster. Although the direction of transfer for alien-to-alien mobilization is indeterminate, the direction of transfer for native-to-alien mobilization could be established. As expected, alien-to-alien mobilization was found much more abundant than the native-to-alien mobilization. The network also highlighted several inter-domain transfers. For example, bidirectional transfers between Euryarchaeota, an archaeal phylum, and several bacterial phyla were revealed; Euryarchaeota was found to be among the highly interactive phyla by both modes of transfer (native-to-alien, alien-to-alien). Among the bacterial phyla, Proteobacteria was found to be most abundant with HGT events, an observation consistent with previous studies [32].

We further emphasize that this unique gene flow network could be realized by invoking the JS-CB method that has been validated using both artificial chimeric genomes and well-characterized bacterial genomes (e.g. *E. coli* K12) [23,27]. This method was shown to be highly efficient in grouping genes originating from a distinct donor in a recipient genome [23], an attribute that was leveraged in the current study to reconstruct horizontal gene flow among prokaryotic taxa. A recent application entailed identifying pathogenicity and antibiotic resistance islands in methicillin-resistant *Staphylococcus aureus* genomes [27]. Even though not designed to identify genomic islands (GIs) *per se*, JS-CB outperformed frequently used GI prediction methods in identifying known GIs in *S. aureus*. This included the SCC*mec* island that harbours *mecA* gene that is known to be involved in the resistance to methicillin. These validations led us to use JS-CB to quantify horizontal gene flux among prokaryotes. Note that JS-CB in itself does not utilize marker gene information or phyletic pattern assessment. It is based solely on gene composition, specifically, the codon usage biases of genes. Here, we included marker enrichment and phyletic pattern analyses to place even more confidence over JS-CB predictions. Not just the JS-CB clusters with atypical composition and marker enrichment were annotated alien but even those atypical clusters that lacked marker gene enrichment but displayed aberrant phyletic pattern were deemed alien. The latter is particular difficult to identify due to lack or depletion of markers; we found numerous such clusters and a further examination highlighted abundance of genes with metabolic functions in several of these clusters. As an example, one such cluster in the genome of *Pelobacter carbinolicus* DSM 2380 harbours genes that encode proteins such as heat shock and transferases, specifically Heat shock 70 kDa protein, Glycosyltransferase like family 2, maltose O-acetyltransferase activity, Glycosyl transferases group 1, transferase activity, transferring glycosyl groups, Polysaccharide pyruvyl transferase, Hexapeptide repeat of succinyl-transferase, Polysaccharide biosynthesis protein and KR domain protein. Past studies have reported horizontal transfer of such genes, for example, glycosyltransferase genes, which aided in enhancing the metabolic flexibility of the recipient organisms [67–70].

The connectivity of prokaryotic HGT network is governed by a power-law distribution. Such networks have been shown to have scale-free and small-world properties. Scale-free networks display identical properties when any random subset of the complete network is sampled, suggesting that our conclusions should not be strongly affected by an ever-increasing number of genomes. In a small-world network, the average shortest path between any two of its nodes (termed ‘network diameter’) involves traversing only relatively few nodes. This has a profound ecological meaning and strong implications for genome evolution. In the context of the HGT network, a small-world structure means that a substantially beneficial gene appearing in any organism can swing across species barriers and reach any other organism via small number of HGT events. This scenario was apparent in the gene flow network—we found a subset of alien genes representing different functionally categories and present in over 10 phyla. These genes may be thought to be conserved across different phyla, however, they were identified in clusters deemed alien by our multi-pronged approach. This suggests that they were likely acquired as a consequence of HGT and have frequently been mobilized across phyla.

The abundance of alien-to-alien mobilization in the network points to a mobile gene network that is most likely formed by gene flow from a common mobile reservoir and have developed over the course of evolution. The functional analysis identified a large number of alien genes that are frequently associated with mobile genome, as well as many genes with yet unknown functions.

Although we focused on inter-phylum transfers that could be of immense interest because of transfers over such large phylogenetic distances, where there could be strong barriers to transfer and integration of foreign elements, it is well known that phylogenetically proximal organisms (such as those within same genus) exchange genes more frequently. However, because of their compositional similarity, transfers of native genes among them would be difficult to ascertain. Although cluster-level analysis presented here enables characterization of genes of ambiguous characters (by their association with well-characterized genes in a cluster), it may also augment false predictions. Additional biological information, such as genome position, marker genes and phylogenetic signals, can help reduce false positives and false negatives in such cases [8,23]. Amelioration of the composition of acquired genes to that of the recipient genome with the passage of time poses a significant challenge in identifying ancient transfers. Although one can invoke the complementary strengths of phylogenetic approaches to address this issue to an extent, these false negatives may also be identified and reclassified based on the aforementioned additional biological information. Future studies could focus on addressing these issues that are important for deciphering HGT and elucidating horizontal flux within and across different taxa and also for quantifying both ancient and recent transfers.

## 5. Conclusion

We leveraged the strengths of composition-based, marker-enrichment-based, and phylogenetic approaches to reconstruct a high confidence gene exchange network. The network enabled identification of genes mobilized across alien components of the prokaryotic genomes and from native components of donor genomes to the recipient genomes. The latter led to the identification of donor–recipient pairs and thus the direction of the transfer, thereby providing us with a guide map to better understand the prokaryotic evolution. The network established in this study may spur further investigations that could augment our understanding of differential gene flow patterns and provide insights into factors that aid or impede HGT.

## Data Availability

The associated datasets can be accessed online at https://github.com/sohamsg90/Gene-flow-network. The data are provided in the electronic supplementary material [71].
